# Homo- and Heterosubtypic Immunity to Low Pathogenic Avian Influenza Virus Mitigates the Clinical Outcome of Infection with Highly Pathogenic Avian Influenza H5N8 Clade 2.3.4.4.b in Captive Mallards (*Anas platyrhynchos*)

**DOI:** 10.3390/pathogens12020217

**Published:** 2023-01-30

**Authors:** Karolina Tarasiuk, Anna Kycko, Edyta Świętoń, Łukasz Bocian, Krzysztof Wyrostek, Krzysztof Śmietanka

**Affiliations:** 1 National Veterinary Research Institute, Department of Poultry Diseases, Al. Partyzantów 57, 24-100 Puławy, Poland; 2 National Veterinary Research Institute, Department of Pathology, Al. Partyzantów 57, 24-100 Puławy, Poland; 3 National Veterinary Research Institute, Department of Epidemiology and Risk Assessment, Al. Partyzantów 57, 24-100 Puławy, Poland

**Keywords:** highly pathogenic avian influenza, low pathogenic avian influenza, mallards, susceptibility, immunity

## Abstract

In this study, we investigated the clinical response, viral shedding, transmissibility, pathologic lesions, and tropism of HPAIV Gs/Gd H5N8 subtype (clade 2.3.4.4b), following experimental infection of three groups of captive mallards (*Anas platyrhynchos*): (i) fully susceptible, (ii) pre-exposed to low pathogenic avian influenza virus (LPAIV) H5N1 subtype, and (iii) pre-exposed to LPAIV H3N8 subtype. Infection of naïve mallards with HPAIV H5N8 resulted in ~60% mortality, neurological signs, abundant shedding, and transmission to contact ducks, who also became sick and died. High amounts of viral RNA were found in all collected organs, with the highest RNA load recorded in the brain. The IHC examinations performed on tissues collected at 4 and 14 days post-infection (dpi) revealed tropism to nervous tissue, myocardium, respiratory epithelium, and hepatic and pancreatic cells. The mallards pre-exposed to LPAIV H5N1 and challenged with HPAIV H5N8 were asymptomatic and showed a significant reduction of viral RNA shedding, yet still sufficient to cause infection (but no disease) in the contact ducks. The AIV antigen was not detected in organs at 4 and 14 dpi, and microscopic lesions were mild and scarce. Similarly, mallards previously inoculated with LPAIV H3N8 remained healthy after challenge with HPAIV H5N8, but viral RNA was detected in large quantities in swabs and organs, particularly in the early phase of infection. However, in contrast to mallards from group I, the IHC staining yielded negative results at the selected timepoints. The virus was transmitted to contact birds, which remained symptomless but demonstrated low levels of viral RNA shedding and mild- to moderate tissue damage despite negative IHC staining. The results indicate that naïve mallards are highly susceptible to HPAIV H5N8 clade 2.3.4.4b and that homo- and heterosubtypic immunity to LPAIV can mitigate the clinical outcomes of infection.

## 1. Introduction

The role of wild aquatic birds in the epidemiology of highly pathogenic avian influenza (HPAI) of Gs/Gd lineage has evolved from sporadic “dead-end” hosts [1,2], through short- and long-distance spreaders with limited duration of virus circulation in the infected population [3,4], to long-distance vectors and reservoirs of the virus that are able to be maintained endemically in resident wild bird populations [5]. This significant shift in host–pathogen interaction is most likely associated with the unprecedented propensity of the recent HPAI virus (HPAIV) H5 subtype (especially clade 2.3.4.4b) to generate a wide range of genotypes in which genetic segments are frequently exchanged with low pathogenic avian influenza viruses (LPAIV) circulating in wild aquatic birds [6]. The acquisition of internal genes from avian influenza viruses that have coevolved with their hosts for a long time may have provided a fitness advantage to the novel HPAIV reassortants, as a result of more efficient replication and transmission. It is worth noting that most of the nine existing neuraminidase (NA) subtypes have formed combinations with H5 clade 2.3.4.4b HPAIV, but only two of them (H5N1 and H5N8) have the particular ability to spark large-scale epidemics in wild birds (and consequently in poultry) [7,8,9]. As an example, during the 2020/2021 and 2021/2022 HPAI seasons, the number of outbreaks caused by these two subtypes in wild birds and poultry in Europe exceeded 5000 and 3500, respectively (data from the European Reference Laboratory for Avian Influenza, available at https://www.izsvenezie.com/reference-laboratories/avian-influenza-newcastle-disease/europe-update/, accessed on 2 September 2022) but the number of outbreaks in wild birds is almost certainly underestimated [7].

The changing epidemiology of HPAI in wild birds calls for more research focused on better understanding which bird species can contribute to long-distance dispersal, local spreading, and maintenance of a virus in a given area. Moreover, it is also important to know why a virus that is defined as highly lethal for some individuals can be relatively benign for other representatives of the species. It is known that “pathogenicity” is the result of complex host–pathogen interplay, in which the inherent high virulence of the virus can be significantly reduced (or even abrogated) by the immune status of the host (e.g., as a result of previous infection or vaccination) and vice versa; low pathogenic viruses can induce a violent clinical course in immunosuppressed individuals or in the presence of co-infections [10,11].

Mallards (*Anas platyrhynchos*) are the most abundant representative of the Anseriformes order and constitute an important reservoir hosts for LPAIV [12]. As the populations of migratory and resident mallards comingle, they play an important role in both the introduction and maintenance of AIV in a given area [13]. Although HPAIVs have been also detected in mallards during the recent HPAI epidemics in Europe, the number of cases reported through passive surveillance (i.e., testing dead or moribund birds) was much lower in comparison with, e.g., geese, swans, or terns [7,14,15]. Experimental infections of mallards with HPAIV H5Nx (Gs/Gs lineage) provided evidence that clinical manifestations in susceptible birds can range from none to severe neurological signs accompanied by mortality [16,17,18]. However, pre-exposure of mallards to LPAIV followed by infection with HPAIV H5 alleviated clinical symptoms but did not abrogate shedding, thus making them potential candidates for transmission of the virus to susceptible populations [19,20].

The objective of our study was to evaluate the clinical outcome, virus replication, transmissibility, histopathological lesions, tropism, and seroconversion following infection with HPAIV H5N8 subtype (Gs/Gs lineage) clade 2.3.4.4b (predominant in the HPAI 2016/17 season) in three groups of captive mallards: (i) naïve, (ii) pre-exposed to LPAIV H5N1, and (iii) pre-exposed to LPAIV H3N8.

## 2. Materials and Methods

### 2.1. Viruses

All avian influenza viruses used in the study were accessed from the repository of the National Veterinary Research Institute, Pulawy, Poland. The HPAIV used for inoculation of birds, A/herring gull/Poland/MB082B/2016 (H5N8) clade 2.3.4.4.b, was detected in Poland during the HPAI epidemic in the 2016/2017 season. Two LPAIV, both isolated from healthy mallards, were also used: A/mallard/Poland/141/2015 (H5N1) and A/mallard/Poland/358/2006 (H3N8). The viruses were titrated in 9 to 11 day-old specific pathogen-free (SPF) embryonated chicken eggs (Valo Biomedia, Osterholz-Scharmbeck, Germany), and the titers were expressed as mean embryo infectious doses (EID_50_/mL).

### 2.2. Birds

The mallards used in the study were hatched in the Game Breeding Center “Gola” in Poland. At 4 days of age, the birds were transported to the biosafety level-3 (BSL-3) animal facility of the National Veterinary Research Institute, Pulawy, Poland, and placed freely in rooms equipped with water tanks for bathing and provided with feed and water ad libitum. Mallards were acclimated until they were 6 weeks old (group I) or 4 weeks old (group II and III). During the acclimatization period, a series of laboratory tests (rRT-PCR and/or ELISA) were carried out on oropharyngeal swabs, cloacal swabs, and fecal- or blood samples, to confirm or rule out the presence of avian influenza virus, rota-, parvo-, corona- and astroviruses, *Salmonella* spp., and common parasites (AIV methodology below; other methodologies available upon request).

### 2.3. Animal Experiments

Three separate experiments were conducted in birds divided into 3 groups (I–III), with 15 mallards in each group.

Group I: Twelve 6-week-old mallards were infected intraocularly and intranasally with HPAIV A/herring gull/Poland/MB082B/2016 (H5N8) at the dose of 10^6^ EID_50_/bird in 0.1 mL. Three contact ducks were placed in the same room 24 h later.

Group II: Twelve 4-week-old mallards were infected intraocularly and intranasally with LPAIV A/mallard/Poland/141/2015 (H5N1) at the dose of 10^6^ EID_50_/bird in 0.1 mL. Two weeks later (i.e., at 6 week of age), the ducks were infected with HPAIV A/herring gull/Poland/MB082B/2016 (H5N8) (infection route, virus dose, and volume as in group I). Three contact ducks were placed in the same room after 24 h.

Group III: Twelve 4-week-old mallards were infected intraocularly and intranasally with LPAIV A/mallard/Poland/358/2006 (H3N8) at the dose of 10^6^ EID_50_/bird in 0.1 mL. Two weeks later (i.e., at 6 week of age), the ducks were infected with HPAIV A/herring gull/Poland/MB082B/2016 (H5N8) (infection route, virus dose, and volume as in group I). Three contact ducks were placed in the same room after 24 h.

Mallards in all groups were observed for 14 days post-infection (dpi) with LPAIV and/or HPAIV. At 2, 4, 7, 10, and 14 days after infection with HPAIV (in case of contact ducks: 1, 3, 6, 9, and 13 days post-contact (dpc)), oropharyngeal and cloacal swabs were collected from all birds, placed in viral transport medium (COPAN, Italy), and stored at −80 °C until further use (viral RNA quantification by real time RT-PCR). Additionally, 4 and 14 days after HPAIV infection, organ samples (brain, trachea, lung, heart, liver, spleen, kidney, pancreas, proventriculus, dudodenum, ileum) from 2 dead or euthanized ducks (4 dpi) or all birds euthanized after termination of the experiment (14 dpi) were collected for viral RNA quantification, histopathology, and immunohistochemistry. Blood samples were also collected at 14 dpi with LPAIV and HPAIV for the purpose of serological testing.

### 2.4. Viral RNA Quantification by Quantitative Reverse Transcription Polymerase Chain Reaction (qrRT-PCR)

The viral RNA was quantified in oropharyngeal swabs (OS), cloacal swabs (CS), and selected organ samples collected from HPAIV-infected mallards by quantitative real time RT-PCR (qrRT-PCR). For the quantifications, a standard curve was established by testing a series of ten-fold dilutions of a sample containing a known amount of the M gene copies. Organ samples were prepared as 20% suspensions by homogenizing 2 g of organ in phosphate buffered saline in a total volume of 10 mL. The homogenates were clarified using centrifugation (3000× *g*, 10 min), and the supernatant was used in the subsequent steps. The RNA was extracted from 200 µL of organ homogenate, swab fluid, or standard curve dilution using a Viral Mini Kit Plus (Syngen, Wrocław, Poland) based on the manufacturer’s protocol. For the detection and quantification of viral RNA load, a QuantiTect Probe RT-PCR Kit (Qiagen, Hilden, Germany) was used with primers and probe targeting the influenza A virus M gene [21]. Based on the standard curve, the number of gene copies per 0.2 mL of swab medium or organ homogenate was calculated for each sample.

### 2.5. Histopathological and Immunohistochemical Examinations

For the histopathological and immunohistochemical examination, tissue samples collected from the brain, trachea, lung, heart, liver, spleen, proventriculus, pancreas, duodenum, ileum, and kidney were fixed in 10% neutral buffered formalin. The tissues were then routinely processed and embedded in paraffin. For histopathology, the paraffin blocks were cut on microtome into 4 µm-thick sections, which were placed on standard glass slides and stained with haematoxylin and eosin (H&E). For immunohistochemistry, the tissue sections cut from the paraffin blocks were placed onto Superfrost glass slides (Menzel–Glaser, Braunschweig, Germany) and incubated in 37 °C overnight. Next, the sections were deparaffinized, rehydrated in descending ethanol concentrations, and subjected to endogenous peroxidase blocking using 3% solution of H_2_O_2_ (30%) in methanol for 10 min, followed by epitope unmasking using protease K (DAKO, Glostrup, Denmark) for 15 min at room temperature. For the detection of viral antigen, anti-influenza A nucleoprotein monoclonal antibody (HYB 340-05, Statens Serum Institute, København, Denmark) was used (dilution 1:1000, 2 h). The antibody detection was performed using a Dako REAL EnVision Detection System, Peroxidase/DAB, Rabbit/Mouse (K5007, DAKO, Glostrup, Denmark). The sections were counterstained with Mayer’s haematoxylin, dehydrated, and mounted. To confirm the specificity of the staining, sections incubated with PBS instead of the primary antibody were used. The tissues were examined under a light microscope (Axiolab, Zeiss, Jena, Germany) for evaluation of histopathological lesions in the H&E-stained sections and the detection of the immuno labelling of the viral antigen in the IHC-stained ones. For an assessment of histopathological lesions, the semiquantitative scoring system proposed by Landnann et al. [22] was applied.

### 2.6. Serological Testing

Hemagglutination inhibition (HI) assays were performed, as described previously [23], using four hemagglutination units of inactivated antigens prepared from AIV strains A/herring gull/Poland/MB082B/2016 (H5N8) (groups I–III, to test with sera collected 2 weeks post-challenge with HPAIV H5N8), A/mallard/Poland/141/2015 (H5N1) (group II, to test with sera collected 2 weeks post-inoculation with homologous LPAIV), and A/mallard/Poland/358/2006 (H3N8) (group III, to test with sera collected 2 weeks post-inoculation with heterologous LPAIV). HI titers ≥16 (≥4 log_2_) were considered positive. Additionally, all sera collected from ducks: (i) at day 0 (i.e., before the experiment), (ii) 2 weeks post-inoculation with LPAIV, and (iii) 2 weeks post-challenge with HPAIV were tested in blocking ELISA AI Multi-Screen Ab Test (Idexx Europe B.V., Hoofddorp, The Netherlands) based on the protocol recommended by manufacturer.

### 2.7. Statistical Analysis

The Kaplan–Meier method was used to perform a survival analysis using data from experimental groups I, II, and III. Additionally, the survival was compared between groups I and II, as well as I and III, using the chi-square test using the Bonferroni correction and the following tests: Gehan the generalized Wilcoxon test, Cox–Mantel, log-rank, and the Peto and Peto version of the Wicoxon test. Comparison of the viral RNA load in swabs between groups was performed for each type of swab and for each timepoint using a non-parametric Kruskal–Wallis test. The results with *p* < 0.05 were considered as statistically significant.

TIBCO Software Inc. (2017) Statistica (data analysis software system), version 13, was used for the analyses.

## 3. Results

### 3.1. Health Status of Mallards Prior to the Experiments

The results of the laboratory tests for the presence of influenza type A RNA (rRT-PCR) or antibodies (ELISA) were negative in all birds. The presence of RNA of gamma and delta coronaviruses was detected in ducks from group I and II. The remaining laboratory examinations gave negative results.

### 3.2. Clinical Signs, Mortality, and Gross Lesions

Following infection of birds with LPAIV H3N8 or H5N1 no clinical signs were observed. After infection of mallards from group I with HPAIV H5N8, the first clinical signs (depression) occurred on the 1st dpi, and in the following days the clinical condition of the birds sharply worsened and more symptoms were observed: recumbency, lethargy, inappetence, and neurological disorders: head tremors, ataxia, paralysis, opisthotonus, lying on the back, and pedaling movements of legs. From day 7 pi onwards, a gradual improvement of health was observed. Mortality occurred between 3–14 dpi and altogether 7/12 mallards died (including two terminally ill birds that were humanely killed at 4 dpi). All statistical tests used to analyze survival indicated statistically significant difference in survival between groups I vs. II and I vs. III (*p* = 0.012 to *p* = 0.016) (Figure S1). In the contact group, all three birds became sick and died between 4–5 dpc. No overt clinical signs or mortality were observed in mallards from groups II and III after infection with HPAIV H5N8. On post-mortem examination, dead mallards from group I revealed pronounced gross lesions that included congestion in the brain, lungs, trachea, liver, spleen, pancreas, kidneys, duodenum, and caecum; petechial hemorrhages in the heart, liver, and proventriculus; ecchymotic hemorrhages in the liver; necrotic foci in the pancreas; and the presence of large quantities of mucosal exudate in the proventriculus. No obvious lesions were found at necropsy in the birds from group II and III euthanized at 4 and 14 dpi.

### 3.3. Virus Shedding and Detection of Viral RNA in Selected Internal Organs

**Figure 1:**
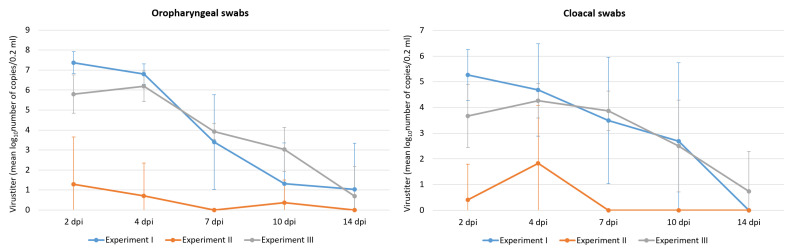
The amount of viral RNA in oropharyngeal and cloacal swabs collected from mallards at 2, 4, 7, 10, and 14 dpi.

**Figure 2:**
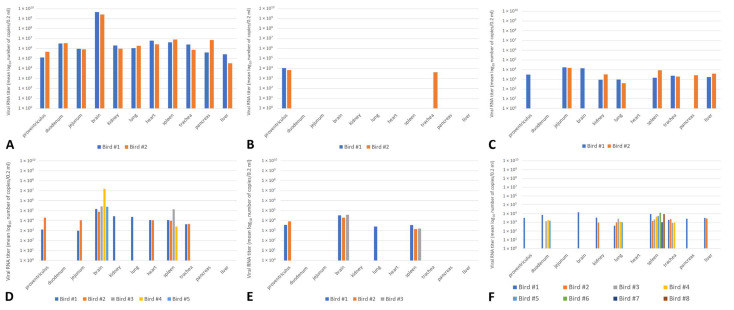
The amount of viral RNA in organs collected from 2 mallards at 4 dpi ((**A**)—group I, (**B**)—group II, (**C**)—group III) and the mallards that survived at 14 dpi ((**D**)—group I, (**E**)—group II, (**F**)—group III).

Following infection with HPAIV H5N8, ducks from all groups shed the virus from both the oropharynx and cloaca, but the duration of shedding and amount of detected viral RNA differed between groups (Figure 1, Table S1). In general, mallards from group I and group III had comparable quantities of viral RNA, and the only statistically significant difference was found in the OS at 2 dpi (group I > group III, *p* < 0.05). In both groups I and III, the obtained mean values of detected RNA reached a peak between 2–4 dpi, with a steady decline in the number of positive birds and level of shedding up to 14 dpi (Figure 1). At 2 and 4 dpi, shedding from the respiratory tract was more pronounced in comparison with the digestive tract in both groups. On the other hand, the mean amounts of RNA detected in group II were very low, and at each timepoint (except 4 dpi) the majority of mallards within the group were negative (Table S1). The level of shedding from OS and CS was statistically significantly higher in group I than group II at 2, 4, and 7 dpi (*p* < 0.05). The statistical comparison of groups II and III revealed significantly higher shedding in birds from group III at 2, 4, 7, and 10 dpi (OS) and at 2, 7, and 10 dpi (CS) (*p* < 0.05). In the contact groups, shedding was detected throughout all the investigated timepoints in mallards from group I, II, and III (Table S1). As for the measurement of the viral RNA load in organs, the highest levels were detected in mallards from group I at 4 dpi (Figure 2, Table S1). All organs were strongly positive in this group, but the highest values (>9log_10_) were detected in the brain. Similarly, all tested organs were also positive in group III at 4 dpi, although the values were lower in comparison with group I. In mallards from group II, only samples of the proventriculus and trachea were weakly positive at 4 dpi. At 14 dpi, the number of birds that tested positive in rRT-PCR varied from 3 to 8, but the number of RNA copies in the tested volume rarely exceeded 4log_10_ (Figure 2, Table S1).

### 3.4. Microscopic Lesions and Immunohistochemical Staining

**Figure 3:**
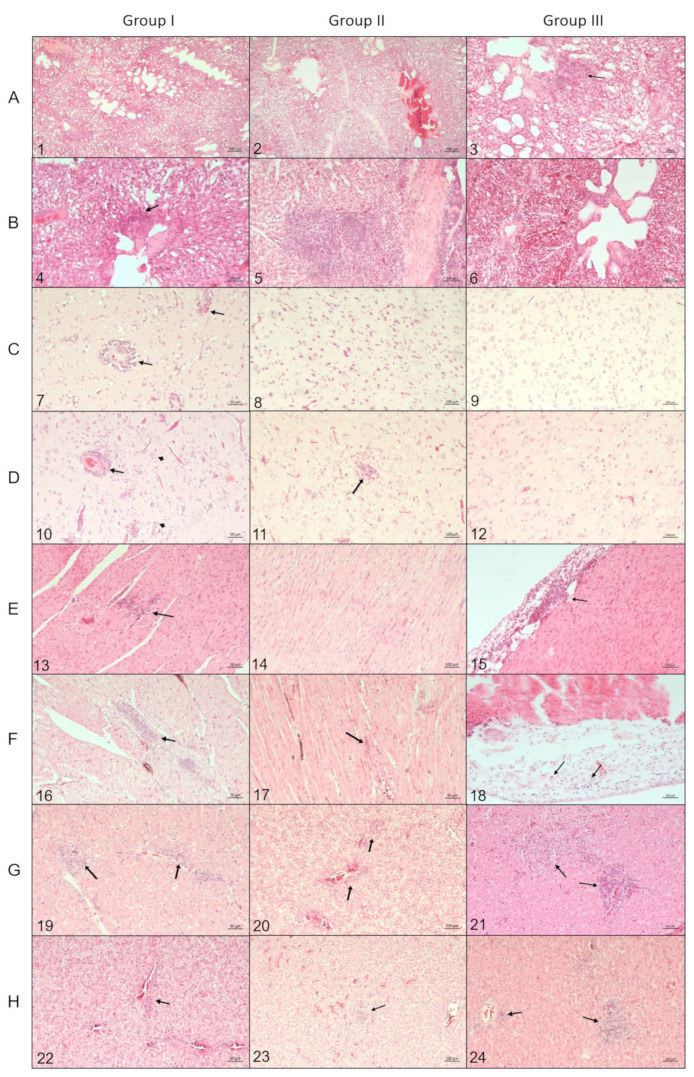
Histopathological changes in the lungs, brain, heart, and liver from the HPAIV-inoculated mallards representing groups I–III at 4 and 14 dpi, example photomicrographs. (**A**) lungs, 4 dpi: no changes in group I, oedema in group II, mild lymphohistiocytic infiltrations around air capillaries (arrow) in group III. (**B**) lung, 14 dpi: lymphohistiocytic infiltrations in group I (arrow), hyperplasia of the peripheral lymphoid tissue in group II, no changes in group III. (**C**) brain, 4 dpi, group I: necrotizing encephalitis, glial nodules, and perivascular lymphocytic infiltrates (arrows); group II: mild gliosis, activated microglia), group III: mild gliosis. (**D**) brain, 14 dpi, group I: mild gliosis (small arrows), perivascular lymphocytic infiltrates (arrow), group II: moderate gliosis, aggregates of microglia around blood vessels, and neurons; group III: mild diffuse gliosis, activated astrocytes. (**E**) heart, 4 dpi, lymphohistiocytic infiltrations (arrow) in the myocardium in group I and within the epicardium in group III, no changes in group II. (**F**) heart, 14 dpi,: lymphohistiocytic infiltrations in the myocardium in the groups I and II edema and mild inflammation within epicardium in group III. (**G**) liver, 4 dpi, groups I–III: interstitial lymphohistiocytic infiltrations (arrows). (**H**) liver, 14 dpi, groups I–III: lymphohistiocytic infiltrations (arrows). Hematoxylin and eosin staining, pictures 1 and 2: objective magnification 5×, pictures 3–24: objective magnification 10×.

In mallards from group I, at 4 dpi, multifocal mild to moderate lymphohistiocytic infiltrations and, rarely, mild parenchymal necrosis were observed in the heart muscle, liver, and brain, where the infiltrations were accompanied by multifocal gliosis (Figure 3 (C7,E13,G19)). In the birds euthanized at 14 dpi, moderate lymphohistiocytic infiltrations were present in the heart, liver, lungs, and around blood vessels in the brain, which also displayed multifocal moderate gliosis (Figure 3 (B4,D10,F16,H22)).

In group II, the birds at 4 dpi displayed only mild lymphohistiocytic infiltrations, visible in the liver (Figure 3 (G20)), whereas those necropsied at 14 dpi showed moderate gliosis in the brain, lymphoplasmatic infiltrates in the liver, heart muscle, and proventricular mucosa, as well as moderate hyperplasia of the peripheral lymphoid tissue in the lungs (Figure 3 (B5,D11,F17,H23)). The changes in the contact ducks at 14 dpi were limited to mild diffuse gliosis in the brain.

In ducks from group III at 4 dpi, moderate lymphohistiocytic infiltrations were found in the liver and heart, particularly around the epicardium (Figure 3 (E15,G21)). At 14 dpi, the birds in this group displayed similar changes in the liver and only minimal ones in the heart and brain (Figure 3 (D12,F18,H24)). In contact birds, in two out of three cases, moderate multifocal gliosis was observed in the brain, and mild to moderate lymphohistiocytic infiltrations in the lung, liver, and heart muscle. The remaining birds had only mild lymphocytic infiltrations in the proventriculus and moderate hyperplasia of the splenic nodules.

No distinctive histopathological changes were found in the pancreas, jejunum, trachea, and kidney in any of the birds from all groups, except singular cases that had mild lymphocytic infiltrates in these organs. In the spleen, in the majority of the 14 dpi ducks in all the groups, mild reticuloendothelial cell proliferation was present.

**Figure 4:**
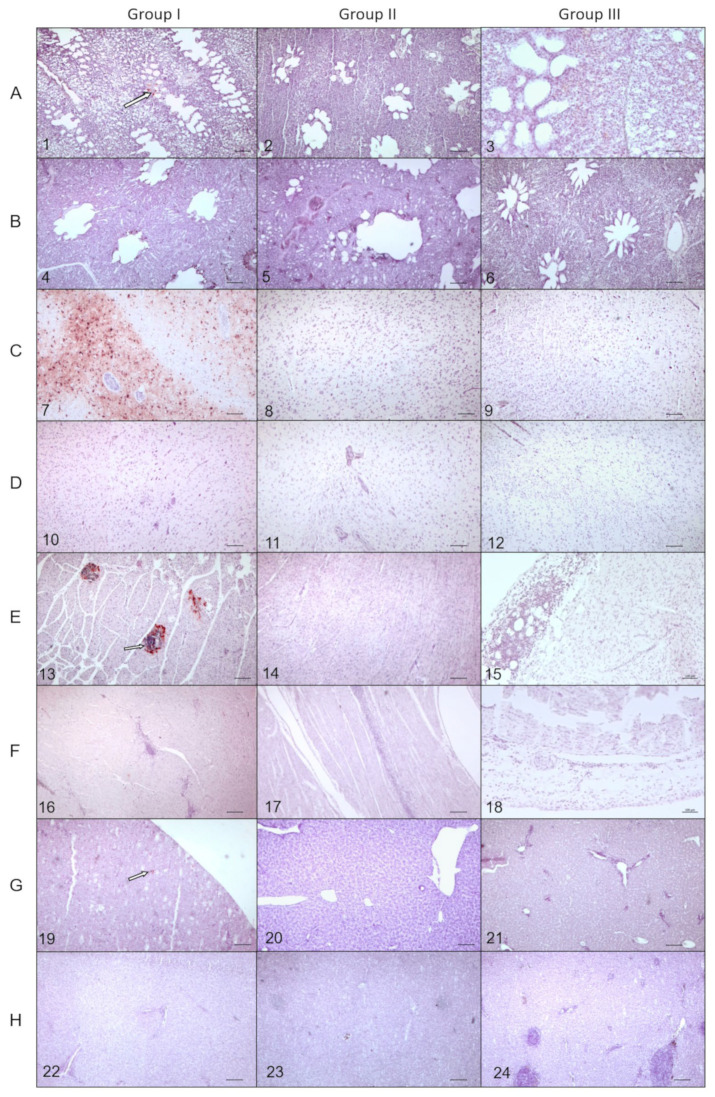
Results of immunohistochemical staining for the presence of AIV antigen in the lungs, brain, heart, and liver from mallards representing groups I-III at 4 and 14 dpi, example photomicrographs; (**A**) lung 4 dpi, (**B**) lung 14 dpi, (**C**) brain 4 dpi, (**D**) brain 14 dpi, (**E**) heart 4 dpi, (**F**) heart 14 dpi, (**G**) liver dpi, and (**H**) liver 14 dpi. Positive IHC reaction, indicating AIV antigen was visible in group I at 4 dpi as dark-brown particles in the lung epithelium (arrow) (**A**), neuronal and glial cells in the brain (**C**), myocytes in the heart (arrow) (**E**), and macrophages within the hepatic sinusoids in the liver (arrow) (**G**). No positive immunolabelling was present in the remaining tissues. Pictures 1, 2, 4, 6–14, 16, 17: objective 5×. Pictures 3, 5, 15, 18–24: objective 10×. Scale bar = 100 µm.

The results of immunohistochemical staining of the tissues collected from ducks in group I at 4 dpi revealed AIV antigen distributed multifocally in the neural and glial cells of the brain (Figure 4 (C7)), respiratory capillaries in the lung (Figure 4 (A1)), myocardial fibers in the heart (Figure 4 (E13)), single parenchymal cells in the pancreas and in the cells that were most likely macrophages within inflammatory infiltrates present in the liver (Figure 4 (G19)), and singular blood vessels in the alimentary tract. Positive immunoreaction was also observed in neurons and glial cells in the brain of 14 dpi-contact-ducks from the same group. There was no positive immunolabelling found at 14 dpi in the tissues from other ducks in this group. The AIV antigen was not detected in any tissues from the birds in the two remaining groups.

### 3.5. Serological Findings

All sera collected prior to the experiments were negative for antibodies against influenza A type. All surviving mallard ducks from group I (*n* = 5) had antibodies to AIV (tested by ELISA) and specifically to H5N8 homologous antigen (measured in HI assay), with an HI geometric mean titer (GMT) of 8.79 log_2_ (range 8–9) (Table 1). Two weeks after inoculation with LPAIV H5N1, all ducks from group II were positive by both ELISA and HI (tested against the homologous H5N1 antigen). The GMT was 5.82 log_2_, and the values of positive titers ranged from 5 to 6 log_2_. The challenge of birds from that group with HPAIV H5N8 resulted in all sera being positive 2 weeks later, and the GMT measured in HI against homologous antigen H5N8 was 6.0 log_2_ (range 4–8 log_2_). Only one serum from a mallard in group III was positive in HI. However, as many as five birds yielded HI titers 3 log_2,_ i.e., close to the positivity threshold of ≥4 log_2_. On the other hand, positive results were obtained in the ELISA assay for seven sera. Post-challenged duck sera from group III had high HI antibody titers (range 7–10 log_2_, GMT = 8.55 log_2_) and they were also positive in the ELISA test.

## 4. Discussion

There is limited information on the virulence of the recent HPAIV H5Nx Gs/Gd lineage to mallards, but a review of the data published so far suggests that pre-exposure to LPAIV modulates the course of infection induced by HPAIV H5Nx [19,20]. Similar results were also published for wood ducks (*Aix sponsa*) [10]. The scientific groups that investigated the aspect of the prior immunity of mallards to LPAIV on the clinical outcome after infection with HPAIV H5 designed two different studies. In the first investigation [20], mallards were inoculated with homo- and heterologous LPAIV (i.e., H5N2 and H4N6) and challenged with HPAIV H5N1 clade 1, one of the earliest Gs/Gd strains and genetically distant from the currently predominant H5 clade 2.3.4.4b viruses. In the second published study [19], the challenge virus HPAI H5N8 belonged to clade 2.3.4.4b (predominant in recent years in Europe) but the authors used mallards naturally exposed to different subtypes of LPAIV, namely H1N3, H3N8, H4N6, H5N3, and H11N9, with an unknown duration of exposure to natural LPAI, hence they were unable to ascertain the immunity status of the birds.

In the study presented here, we performed all our investigations under an experimental setting, and as a challenge virus we used the HPAIV H5N8 subtype clade 2.3.4.4b that predominated in Europe during the epidemic season 2016/17 [15]. The LPAIV strains used for pre-exposure of mallards were deliberately selected in such a way that the first strain possessed homologous hemagglutinin H5 and heterologous neuraminidase N1, and that the second strain had heterologous hemagglutinin H3 and homologous neuraminidase N8. It is noteworthy that the LPAIV H3N8 subtype is prevailing in wild waterfowl, particularly mallards [12,24]. As we did not have access to specific-pathogen-free ducks, we used healthy ducks obtained from a commercial source, for which a number of laboratory tests aimed at detection of common pathogens were carried out. In groups I and II, we detected the RNA of gamma and delta coronaviruses. As the circulation of coronaviruses in apparently healthy populations of wild and domestic ducks have been described [25,26], we assumed that the interfering effects of these viruses on the ducks’ susceptibility to subsequent avian influenza virus infection would be minimal. However, the confidence level of this assumption is low, since there are no published results exploring the potential interaction between corona- and influenza viruses in ducks.

The challenge HPAIV H5N8 clade 2.3.4.4b virus turned out to be highly lethal for fully susceptible mallards, causing a violent clinical course with neurological disorders and ~60% mortality. These findings are in line with previous research, in which the HPAIV H5 Gs/Gd lineage was highly lethal to non-exposed mallard ducks and Pekin ducks, which are domesticated forms of *Anas platyrhynchos* [19,20,27]. The virus was shed in large quantities from respiratory and digestive tracts, particularly during the first week after challenge, and was rapidly transmitted to susceptible individuals, who also became sick and died. Histopathological lesions in this group commonly involved lymphoplasmatic encephalitis, hepatitis, myocarditis, and, to a lesser extent, interstitial pneumonia, and the changes were generally more severe and pronounced than in the birds of other groups. The IHC results indicated H5N8 virus predilection to the nervous tissue, myocardium, respiratory epithelium, and hepatic and pancreatic cells. The virus was also detected in the inflammatory cells, which had characteristics of macrophages. Similar histopathological lesions and viral antigen distribution were previously reported in Pekin ducks infected with HPAIV H5N8 by Pantin-Jackwood et al. [28] and Stoute et al. [29]. The results of this subset of experiments indicate that, despite the high mortality, a fraction of HPAIV-infected mallards can survive at least 14 days post-infection and still remain rRT-PCR-positive. Thus, they can potentially contribute to the contamination of the local environment but their involvement in virus dispersal at a larger geographical scale needs clarification, due to the high morbidity that could negatively impact on their movement patterns.

Mallards in group II showed no HPAIV H5N8-induced morbidity or mortality during the 14-day observation period and expressed low levels of viral RNA in both respiratory and digestive tracts. Additionally, the mild microscopic lesions and absence of AIV antigen in the tested timepoints indicates a high level of protection in comparison with group I, which is most plausibly explained by the interference of homologous immunity to AIV H5. Nevertheless, the level of modulation is quite surprising, considering that both viruses shared only a 89.3% similarity at HA protein level. Moreover, although a heavy reduction in HPAIV shedding was observed in infected mallards, the virus made its way to contact birds, which despite the absence of disease were rRT-PCR-positive, and one of them showed some lesions in the brain. Therefore, the role of HPAIV-infected mallards with homologous immunity as potential contributors to the local perpetuation of the virus is most likely minor but cannot be completely ruled out.

All ducks from group III survived infection with HPAIV H5N8 and remained healthy throughout the experiment, despite a poor antibody response prior to challenge with HPAIV H5N8. This finding can be explained by the pre-existing innate immunity stimulated by LPAIV infection and/or the contribution of cell-mediated immunity. In particular, the impact of the homologous neuraminidase subtype present in LPAIV H3N8 could have contributed to the mitigation of infection outcomes following challenge with HPAIV H5N8, although the homology at amino acid level between NA of H3N8 and H5N8 viruses was only 91.8%. All these aspects require further investigation in future studies. Similarly to ducks in group I, the birds in group III exhibited a high level of HPAIV RNA in swabs and organs, but in contrast to group I, no AIV antigen was detected in organs at 4 dpi and 14 dpi. Interestingly, despite the successful transmission to contact ducks (as evidenced by the detection of shedding), none of them became sick or died, although some birds developed microscopic lesions in the brain, lung, heart, and liver. The most likely explanation for this is that the amounts of virus shed by the infected ducks in the early phase of infection were sufficient to induce some level of tissue damage in contact birds but were still below the threshold dose that is necessary to induce clinical disease and mortality, as observed in ducks in group I, which excreted significantly higher amounts of the virus in the first days post-infection than ducks in group III. Nonetheless, the results of our investigations in group III raise important epidemiological concerns, as apparently healthy but actively-infected mallards could potentially disseminate the virus and/or contribute to local environmental contamination. Taking into consideration the high prevalence of LPAIV H3N8 in the population of wild waterfowl hosts [12], the number of HPAIV H5N8 asymptomatic shedders among mallards might have been underestimated.

To summarize, our results contribute to a better understanding of the complex nature of the host–pathogen interactions in mallards with- or without homo- and heterosubtypic immunity to avian influenza virus. The clinical response following HPAIV infection can vary, from asymptomatic in mallards with previously acquired immunity, to severe disease in naïve birds. The clinical outcome of HPAI in LPAIV-exposed birds is likely dependent on the time that elapsed between infection with LPAIV and HPAIV (in our study: 2 weeks); one can expect that the immunity acquired as a result of exposure to LPAIV wanes with time. Due to the fact that the duration of LPAIV-induced immunity is largely unknown and, in all likelihood, varies between virus strains, it is hard to predict when birds infected with low pathogenic virus will become susceptible to HPAIV again. It is also difficult to forecast how translatable these results are to other LPAIVs; this aspect requires further investigation. Nonetheless, mallards can be considered as a target species for both passive and active HPAI surveillance. In particular, active surveillance can contribute to a better understanding of the holistic picture of the epidemiological situation in wild birds. Moreover, asymptomatically infected mallards can shed the HPAI virus, thus contributing to its transmission to other birds and contamination of the environment.

## Figures and Tables

**Table 1:** Serological status of mallards: (i) non-exposed to AIV (group I); (ii) inoculated with LPAIV H5N1 and challenged by HPAIV H5N8 (group II); and (iii) inoculated with LPAIV H3N8 and challenged by HPAIV H5N8 (group III).

	Day 0		2 Weeks after Inoculation with LPAIV		2 Weeks after Challenge with HPAIV	
	HI	ELISA	HI	ELISA	HI	ELISA
Group I	nt *	neg	n/a **	n/a	5/5 (8.79 log_2_)	5/5
Group II	nt	neg	12/12 (5.82 log_2_)	12/12	10/10 (6.0 log_2_)	10/10
Group III	nt	neg	1/12 (2.37 log_2_)	7/12	10/10 (8.55 log_2_)	10/10

* not tested, ** not applicable.

## Data Availability

Raw data are available upon request from the corresponding author.

## Supplementary Materials

The following supporting information can be downloaded at: https://www.mdpi.com/article/10.3390/pathogens12020217/s1, Table S1: The amount of viral RNA in swabs and organs from mallards (individual results). Figure S1: The Kaplan–Meier analysis of survival in three groups of mallards infected with HPAIV H5N8 (group I), LPAIV H5N1 followed by HPAIV H5N8 (group II) and LPAIV H3N8 followed by HPAIV H5N8 (group III).
