# Supplementary figures and images for: Homo- and Heterosubtypic Immunity to Low Pathogenic Avian Influenza Virus Mitigates the Clinical Outcome of Infection with Highly Pathogenic Avian Influenza H5N8 Clade 2.3.4.4.b in Captive Mallards (Anas platyrhynchos)
Source: Pathogens. 2023 Jan 30;12(2):217. doi: 10.3390/pathogens12020217 (PMC9964785; doi:10.3390/pathogens12020217)

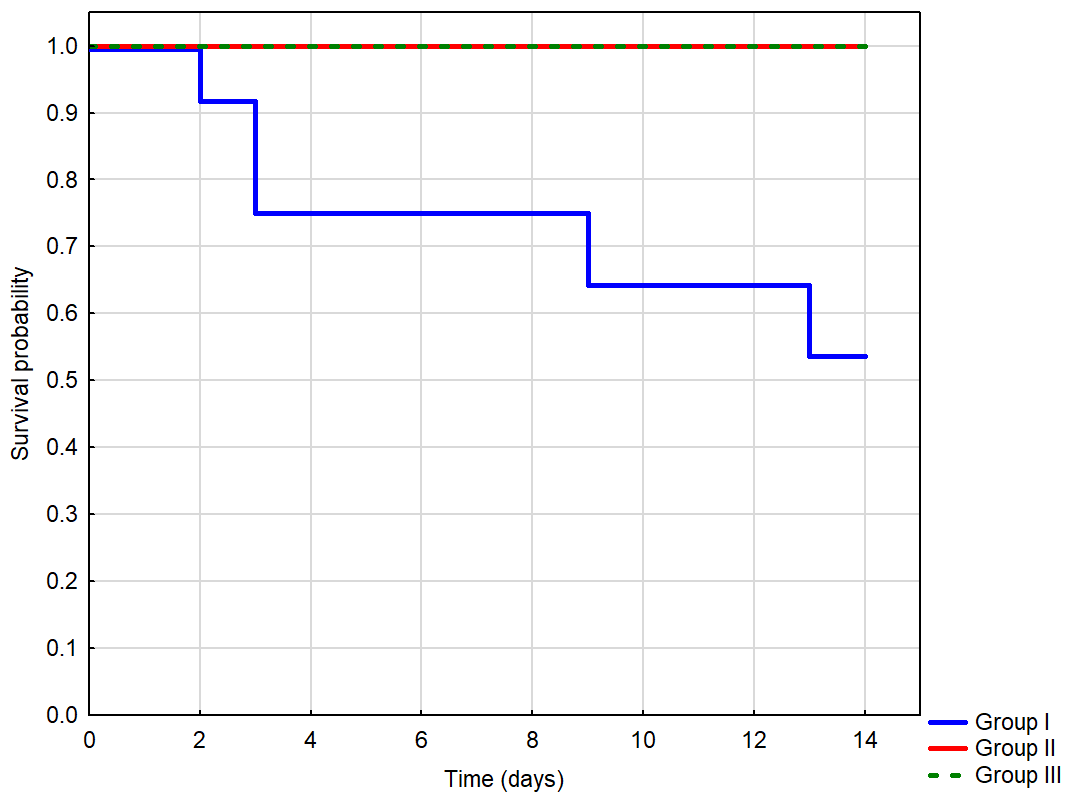

Supplement: Supplementary file 1 [file pathogens-12-00217-s001.zip › pathogens-2131962-supplementary material-Figure S1-1.png]
